# Emergence of New Delhi Metallo-β-Lactamase, Austria

**DOI:** 10.3201/eid1701.101331

**Published:** 2011-01

**Authors:** Gernot Zarfel, Martin Hoenigl, Eva Leitner, Helmut J.F. Salzer, Gebhard Feierl, Lilian Masoud, Thomas Valentin, Robert Krause, Andrea J. Grisold

**Affiliations:** Author affiliation: Medical University of Graz, Graz, Austria

**Keywords:** Bacteria, New Delhi metallo-beta-lactamase, NDM-1, PCR, Klebsiella pneumoniae, Austria, multiple drug resistance, resistance, expedited, letter

**To the Editor:** Extended-spectrum β-lactamase–producing *Enterobacteriaceae* strains have emerged as a major public health problem throughout the world, particularly in India and Pakistan. The widespread use of carbapenems, the only agents reliably active against these bacteria, resulted in the emergence of a new resistance mechanism. New Delhi metallo-β-lactamase (NDM-1) was first detected in a *Klebsiella pneumonia*e isolate in 2008 from a Swedish patient of Indian origin; it has since been reported in increasing numbers of infections in patients from India, Pakistan, and the United Kingdom (*1**–**3*).

NDM-1 shares very little identity with other metallo-β-lactamase enzymes; *Enterobacteriaceae* isolates with NDM-1 show high resistance to nearly all commonly used antibacterial agents (*4*). Most NDM-1 patients in Europe and the United States had received medical care in India or Pakistan before isolation of the strain. However, the emergence of NDM-1 poses the risk of plasmid-mediated transfer of the carbapenemase enzyme *bla*_NDM-1_ between different bacterial strains, which could lead to serious public health issues (*3**,**5*). We report the emergence of NDM-1–positive *K. pneumoniae* in Austria in 2009–2010.

Primers for PCR detection of NDM-1 were designed according to GenBank (National Center for Biotechnology Information, National Institutes of Health, Bethesda, MD, USA) database entry AB571289.1 (www.ncbi.nlm.nih.gov/nuccore/300422615). The forward primer NDM-1gf 5′-ACC GCC TGG ACC GAT GAC CA-3′ (positions 80–99), and reverse primer NDM-1gr 5′-GCC AAA GTT GGG CGC GGT TG-3′ (positions 343–324) were used.

PCR conditions were the following: initial denaturation at 94°C for 5 min; 35 cycles at 95°C for 30 s, 58°C for 30 s, and 72°C for 30 s; and final incubation for 10 min at 72°C. Taq DNA polymerase and dNTPs from QIAGEN (Hilden, Germany) were used. The 264-bp fragment was sequenced and compared with the GenBank entry for NDM-1.

Carbapenemase-producing *K. pneumoniae* has been detected in 26 isolates obtained during September 2007 through August 2010 from 6 patients at the University Hospital, Graz, Austria. Eight isolates from 2 patients were found to carry the plasmid NDM-1. The first case involving NDM-1 occurred in November 2009, and the second occurred in August 2010. Automated repetitive element PCR, conducted with the DiversiLab system (bioMérieux, Marcy l'Etoile, France) (*6*) showed a genetic relatedness of isolates from the 2 patients of ≤81.1% (5 band differences), which indicated independent clones. Isolated NDM-1 strains exhibited resistance to nearly all antibacterial agents, including aztreonam, ciprofloxacin, and gentamicin, and were susceptible to only colistin, tigecycline, and amikacin (Table).

Patient 1, a 30-year-old Austrian man, was admitted to University Hospital (Graz, Austria) in November 2009. His medical history showed he had experienced multiple open fractures of his upper and lower left leg as well as rectal laceration because of a motorcycle accident in Pakistan. His treatment had taken place primarily in surgery departments in Pakistan and India. During his hospitalization in Austria, multiple resistant gram-negative bacteria were isolated, including highly resistant NDM-1–producing *K. pneumoniae*. The NDM-1 strain was isolated twice, from a sacral decubitus ulcer and from stool. After 5 months of recurrent hospitalizations with various infectious complications, multiple anti-infective regimens, and surgical interventions required to treat fractures resulting from the patient’s motorcycle accident, the patient was released without further medical problems.

In August 2010, patient 2, a 14-year-old boy from Kosovo, was transferred from a hospital in that country to the Department of Pediatrics, University Hospital (Graz, Austria) with multiple intra-abdominal abscesses and peritonitis. He had undergone an appendectomy in Pristina, Kosovo, in April 2010, after which abdominal sepsis developed. His travel history was completely unremarkable. On the day of admission, multiple-drug resistant *K. pneumoniae* was isolated from 5 sites (2 swab samples from the abdominal wound, 1 sample from the throat, 1 sample of secretion from an abdominal fistula, and 1 sample from stool). As of November 2010, the patient still required medical care and remained hospitalized.

Most plasmids with the carbapenemase enzyme *bla*_NDM-1_ were shown to be readily transferable and prone to rearrangement, which indicates a potential to spread among bacterial populations (*3*). So far, NDM-1 carbapenemase has been detected in *K. pneumoniae*, *Escherichia coli*, *Citrobacter freundii*, *Enterobacter cloacae,* and *Morganella morganii* and has shown resistance to nearly all classes of antibacterial agents, except polymyxins and tigecycline (*2**,**3*). Kumarasamy et al. recently reported the identification of 37 isolates with NDM-1 in the United Kingdom. The isolates came from 29 patients, of whom at least 17 had traveled to India or Pakistan in the year preceding identification of NDM-1; 14 patients had been admitted to a hospital in those countries (*2*).

NDM-1 has also been isolated from 3 patients in the United States, all of whom had recently received medical care in India (*7*). In contrast, 1 of the 2 patients with *K. pneumoniae–*carrying NDM-1 reported here was transferred to our hospital from Kosovo in southeastern Europe and had an unremarkable travel history. Immediate action is needed to control the spread of NDM-1 and avoid a worldwide public health problem.

**Table:** Antimicrobial drug susceptibilities of New Delhi metallo-β-lactamase strains isolated from 2 patients, Graz, Austria, 2010*

Drug	MIC, mg/L	
	Patient 1 isolate	Patient 2 isolate
Colistin	0.125	0.125
Tigecycline	2	0.125
Amikacin	8	2

*Only substances for which isolates had susceptibility are listed. MICs were determined by the Etest method (AB BIODISK, Solna, Sweden). Susceptibility was determined according to relevant testing conditions and the new susceptibility interpretation standards proposed by the Clinical and Laboratory Standards Institute (www.clsi.org).
